# Comparison of multiplex PCR capillary electrophoresis assay and PCR-reverse dot blot assay for human papillomavirus DNA genotyping detection in cervical cancer tissue specimens

**DOI:** 10.3389/fpubh.2024.1421774

**Published:** 2024-07-19

**Authors:** Lei Qin, Dan Li, Zhihui Wang, Jianyun Lan, Chunrong Han, Jing Mei, Jianxiang Geng

**Affiliations:** ^1^Nanjing Leiyue Clinical Laboratory, Nanjing, Jiangsu, China; ^2^Department of Pathology, Linyi Cancer Hospital, Linyi, Shangdong, China; ^3^Department of Pathology, Yancheng First People’s Hospital, Yancheng, Jiangsu, China; ^4^Department of Pathology, Nanjing Lishui District People’s Hospital, Nanjing, Jiangsu, China; ^5^Department of Pathology, People’s Hospital of Dangtu, Ma'anshan, Anhui, China; ^6^The Cross-Strait Precision Medicine Association HPV Infection Disease Professional Committee, Nanjing, China

**Keywords:** HPV DNA detection, polymerase chain reaction, cervical cancer tissue specimen, multiplex PCR capillary electrophoresis assay, PCR-reverse dot blot assay

## Abstract

**Background:**

The study aimed to evaluate the positivity rates and genotype distribution of the multiplex PCR capillary electrophoresis (MPCE) and PCR-Reverse Dot Blot (PCR-RDB) assays for human papillomavirus (HPV) detection in cervical cancer tissue specimens, and to explore their detection principles and applications in large-scale population screening.

**Methods:**

The MPCE and PCR-RDB assays were performed separately on 425 diagnosed cervical cancer tissue specimens. Subsequently, the results of both assays were compared based on the HPV infection positivity rates and genotype distribution.

**Results:**

The overall positive rates of HPV genotypes for the MPCE and PCR-RDB assays were 97.9% and 92.9%, respectively. A *p*-value < 0.001 indicated a statistically significance difference in consistency between the two assays. The kappa value was 0.390, indicating that the consistency between both assays was fair. HPV16 was the most common single-genotype infection type, with infection rates detected via MPCE and PCR-RDB assays being 75.7% and 68.3%, respectively. In the age group >50 years, the HPV multiple-type infection rate detected via MPCE assay was significantly higher than that detected by the PCR-RDB assay, with a statistically significant difference (*p* = 0.002).

**Conclusion:**

To reduce the false-negative rate and improve screening efficiency, the MPCE assay, which targets the oncogenic gene E6/E7 segments, can be extended to the general female population for the early detection, diagnosis, and treatment of cervical cancer.

## 1 Introduction

Cervical cancer ranks as the fourth most common malignant tumor threatening the life and health of women worldwide (1). The primary cause of cervical cancer and its pre-cancerous lesions is the persistent infection of oncogenic human papillomavirus (HPV) (2). An epidemiological study indicates that almost all cases of cervical cancer are attributable to HPV (3). However, approximately 5% of tumors are not associated with persistent HPV infection (4). Cervical cancer stands out as the only gynaecological malignant tumor with well-defined etiology globally. It is also unique among malignant tumors in that it can be eradicated through comprehensive tertiary preventive measures.

In 2021, the “WHO guideline for screening and treatment of cervical pre-cancer lesions for cervical cancer prevention” recommends using HPV DNA testing as part of a comprehensive approach to screening, triage, and treatment. This guideline advises regular screening at the age of 30 years, with intervals of 5 to 10 years between screenings (5). In 2022, the latest Cervical Cancer Screening Programme in China included HPV nucleic acid testing as one of the primary screening methods (6). HPV DNA detection can be performed on cervical specimens using signal amplification techniques or nucleic acid amplification through polymerase chain reaction (7). These assays have proven to be more sensitive and efficient than cytology methods. Additionally, they are more objective, as they do not rely on visual inspection expertise (8).

HPV is a closed circular double-stranded DNA virus with a genome length of approximately 8 kilobase pairs (kbp). Its genome encodes early regulatory proteins (E1, E2, E4, E5, E6, and E7), late structural proteins (L1 and L2), and the long control region. The early proteins primarily participate in virus replication and transcriptional regulation, while the late proteins are responsible for forming the virus capsid. The long control region is a regulatory area of the virus closely associated with virus replication and transcriptional processes. Genotypes with genome sequence differences >10% are categorized as different genotypes (9–11). Over 200 HPV genotypes have been identified and classified into High-Risk (HR-HPV) and Low-Risk (LR-HPV) types based on their potential to cause cancer (12). HPV16 and 18 are widely recognized as the predominant HR-HPV types worldwide, while HPV52 and HPV58 are commonly found in Asia (13–15).

The significant geographical variation in cervical cancer rates is largely due to non-existent or inadequate screening in public health care settings, coupled with limited access to standard treatment options. Detecting HPV infection in cervical cancer specimens is considered the gold standard for confirming persistent high-risk HPV infection and establishing the pathogenetic association between HPV infection and cervical cancer. However, the challenge lies in obtaining pathologic tissue specimens, which poses a challenge for scientific research. Formalin-fixed, paraffin-embedded (FFPE) tissue specimens stored in pathology departments worldwide are crucial resources for diagnostic purposes when fresh clinical material is unavailable. They also serve as invaluable resources for retrospective molecular and epidemiological studies, especially when investigating rare clinical conditions where prospective data collection is impractical. The quality of extracted DNA is usually assessed via PCR amplification of housekeeping genes. DNA fragmentation can significantly impair PCR efficiency. Hence, primers that amplify a relatively small fragment of the human genome (<270 bp) when working with FFPE material should be used preferably. An amplifiable internal control ensures successful DNA extraction, the absence of PCR inhibitors, and reliable HPV detection, enabling the performance of HPV genotyping on small molecular fragments in cervical cancer tissues (16).

Screening measures are essential for patients with detected early cervical lesions (17). In China, the predominant techniques for HPV nucleic acid detection rely on nucleic acid amplification and its derivatives, which are effective in detecting the entire HPV genome or a specific segment of it (18, 19). In recent years, nearly 100 different HPV assays have become commercially available, varying in the principles, types, and number of HPV genotypes they detect, as well as their clinical utility. Target region selection has a significant impact on an HPV DNA test’s ability to detect cancers. The majority of assays target the L1 region of the virus, and the gene is prone to deletion during virus integration as the disease progresses (20). Therefore, selecting an appropriate and validated test for clinical accuracy, reproducibility, and cost-effectiveness is crucial before implementing screening programs (21–23). This study aimed to assess the diagnostic value of two commonly used HPV DNA genotyping assays: the Multiplex PCR Capillary Electrophoresis (MPCE; targeting E6/E7 segments) and PCR-Reverse Dot Blot (PCR-RDB; targeting L1 segment) assays. McNemar’s test was performed to compare the consistency of genotyping results of both assays in cervical cancer tissue specimens (CCTS) from 425 patients with cervical cancer who underwent surgery. The findings of this study could help to facilitate the selection of appropriate HPV nucleic acid detection reagents and systems based on national cervical cancer prevention and control strategies, laboratory requirements, consideration methodology, different detection targets and types, automation levels, and accessibility. This selection could provide a theoretical basis for the strategy of ‘HPV vaccination with HPV nucleic acid detection-based cervical cancer screening’ in the Chinese female population.

## 2 Materials and methods

### 2.1 Samples

Overall, 425 samples of FFPE tissue specimens were selected from patients diagnosed with cervical cancer by the pathology department at four hospitals in eastern China, spanning from August 2021 to the end of July 2023 (Supplementary Table S1). Two experienced pathologists reviewed the samples according to the classification standards for gynecological tumors (24). They examined the FFPE cervical cancer tissue sections and the clinical pathology data of the patients to ensure the quality of the cancer tissue samples. The age of patients ranged from 21 to 88 years old, with an average age of 54.69 ± 11.92 years. Simultaneously, patient demographic data were collected. This study was approved by the ethics committee of the hospitals, and all participants provided informed consent.

### 2.2 DNA extraction from FFPE cervical cancer tissue specimens

The QIAamp DNA FFPE Tissue Kit (Catalog Number: 56404, QIAGEN GmbH, Germany) was used to purify genomic DNA from FFPE tissue sections. Firstly, the excess paraffin around each paraffin-embedded tissue was removed and each paraffin-embedded tissue was cut into 5 μm thick slices, yielding for 5–8 slices. The tissue pellet was resuspended in 180 μL Buffer ATL buffer and 20 μL proteinase K, then incubated at 56°C for 1 h, to partially reverse the formaldehyde modification of DNA, the samples were then incubated at 90°C for 1 h. After brief centrifugation, we added 2 μL RNase A (100 mg/mL) and incubated samples at room temperature for 2 min to avoid RNA contamination. In the following two steps, AL buffer and ethanol were consecutively added to samples and vortexed thoroughly. Next, we transferred the entire lysate to the QIAamp MinElute column and placed it in a 2 mL collection tube. After centrifugation, we placed the QIAamp MinElute column in a clean 2 mL collection tube. The nucleic acid was adsorbed to the membrane of the QIAamp MinElute column and then washed by AW1 and AW2 buffer. Finally, 50 μL ATE buffer was added to the center of the membrane. After incubating at room temperature for 5 min, the samples were centrifuged at 14,000 rpm for 2 min, and the DNA was collected into new sterile 1.5 mL micro-centrifuge tubes. The Experiment was performed according to the QIAamp DNA FFPE Tissue Kit manufacturer’s handbook.

### 2.3 HPV-DNA genotyping test

Both MPCE and PCR-RDB assays were employed separately to detect and obtain HPV genotyping results from the 425 DNA samples. Table 1 shows the characteristics of the two PCR HPV-DNA genotyping assays: MPCE and PCR-RDB assays. The MPCE and PCR-RDB assays targeted E6/E7 and L1, respectively. Table 1 shows that the MPCE assay can detect one additional HR-HPV26 type and one additional LR-HPV44 type compared to the PCR-RDB assay.

**Table 1:** Characteristics of the two PCR HPV-DNA genotyping assays.

Assay	Type of technology	Gene targeted	HPV DNA genotyping	Internal quality control
MPCE	Multiplex PCR capillary electrophoresis	E6/E7	HR-HPV: 16, 18, 26, 31, 33, 35, 39, 45, 51, 52, 53, 56, 58, 59, 66, 68, 73, 82	Human b-globin gene
				And
			LR-HPV: 6, 11, 42, 43, 44, 81, 83	PcDNA
PCR-RDB	PCR-Reverse Dot Blot	L1	HR-HPV: 16, 18, 31, 33, 35, 39, 45, 51, 52, 53, 56, 58, 59, 66, 68, 73, 82	β-actin gene segment
			LR-HPV: 6, 11, 42, 43, 81, 83	

#### 2.3.1 MPCE assay

HPV genotyping was conducted on 425 samples using the MPCE assay (Ningbo HEALTH Gene Technologies Co., Ltd. China). The SureX® HPV 25X Genotyping Kit (Catalog Number: 06937044500040), Real-time PCR System SLAN-96P (Fosun Diagnostics (Shanghai) Co., Ltd. China), and CE2400 Automated Capillary Electrophoresis Analyzer (Ningbo HEALTH Gene Technologies Co., Ltd. China) were employed to detect HPV targeting the carcinogenic E6/E7 gene segments, allowing for specific amplification and capillary electrophoresis separation of genes from different HPV genotypes. The PCR program included an initial denaturation step at 42°C for 5 min and 95°C for 8 min, followed by 35 cycles of 94°C for 30 s, 60°C for 30 s, 70°C for 60 s, with a final extension at 70°C for 1 min. Internal reference controls, pcDNA, and human genomic reference β-globin were used to monitor the PCR reaction process and sample handling, respectively. The detection procedure strictly adhered to the instructions provided in the reagent kit and detection system manual. The testing process was fully automated and integrated.

#### 2.3.2 PCR-RDB assay

HPV genotyping was performed on 425 samples using PCR-RDB assay (Yaneng Bioscience, Shenzhen, China). The HPV Genotyping Test Kit (Catalog Number: 210608), Real-time PCR System SLAN-96P (Fosun Diagnostics (Shanghai) Co., Ltd. China), and HPV Genotyping Gene Chip Detection System (Yaneng BIOscience Shenzhen Co., Ltd.) were used to detect 23 HPV genotypes, including 17 HR HPV and 6 LR HPV genes, targeting L1 segment (Table 1). The detection process was conducted following the manufacturer’s instructions on the test kit and detection system, including DNA extraction, amplification, hybridization, membrane washing, and data interpretation. The PCR program included an initial denaturation step at 50°C for 15 min 95°C for 10 min, followed by 10 cycles of 94°C for 10 s, 42°C for 90 s, 72°C for 30 s. This was followed by 30 cycles of 94°C for 10 s, 46°C for 60 s, and 72°C for 20 s, with a final extension at 72°C for 5 min. Interpretation was conducted based on the specific HPV genotype sites on the chip. Negative and positive controls were included throughout the experiment. Simultaneously, internal and external quality evaluations were conducted to ensure that the results met the requirements of the laboratory.

### 2.4 Statistical analysis

A paired design χ2 test (McNemar’s)was performed to compare the positivity rates of the MPCE and PCR-RDB assays with the gold standard (pathological histological diagnosis results). The kappa value was used to assess the consistency between both assays. The positivity rates of two different HPV genotyping assays were analyzed in CCTS, exploring the differences in positivity rates between both assays and their corresponding pathological basis. The chi-square and Fisher’s exact tests were used to assess association and compare genotypes by age. Statistical significance was set at *p* < 0.05. All statistical analyses were performed using SPSS 19.0 (IBM Corp., Armonk, NY, United States).

## 3 Results

### 3.1 Analysis of the results obtained from the two assays

Table 2 shows that among the total 425 CCTS, the MPCE assay detected nine negative results, accounting for 2.1% (9/425), while the PCR-RDB assay detected 30 negative results, accounting for 7.1% (30/425). There were eight cases in which neither assay detected HPV infection. The overall positive detection rates of HPV genotypes for the MPCE and PCR-RDB assays were 97.9% and 92.9%, respectively, both >90%. This suggests that both assays demonstrated relatively high overall positive rates for HPV detection in CCTS. A *p*-value < 0.001 indicated a statistical difference in consistency between both assays. Agreement between the tests was assessed based on the kappa value. Kappa values range from 0.81–1.00, indicating almost perfect agreement, 0.61–0.80 substantial agreement, 0.41–0.60 moderate agreement, 0.21–0.40 fair agreement, and 0.00–0.20 slight agreement. The kappa value was 0.390, indicating that the consistency between both assays was fair.

**Table 2:** Overall positive rates of HPV DNA between the two assays.

		MPCE			McNemar test	Kappa value
		Positive	Negative	Total		
		*N* (%)	*N* (%)	*N* (%)		
PCR-RDB	Positive	394(94.7)	1(11.1)	395(92.9)	*p* < 0.001^*^	0.390
	Negtive	22(5.3)	8(88.9)	30(7.1)		
	Total	416(97.9)	9(2.1)	425(100.0)		

^*^A *P*-value < 0.001 indicated a statistical difference in consistency between the two assays. The kappa value is 0.390 indicated that the consistency between the two assays was fair.

### 3.2 HPV genotyping distribution in HPV-positive CCTS

Table 3 presents the distribution of the top nine high-frequency single HPV genotype infections and mixed infections detected via both assays in CCTS. The results showed that the positive rates of single-type HPV-16 infection detected via the MPCE and PCR-RDB assays were 75.7% and 68.3%, respectively, in CCTS. Furthermore, the positive rates of single-type HPV-18 infection detected through the MPCE and PCR-RDB assays were 9.9% and 11.8% in CCTS, respectively.

**Table 3:** HPV genotyping distribution in HPV-positive cervical cancer tissue specimens.

	Type	MPCE	Frequency	PCR-RDB	Frequency	χ2 Test^*^	*P*
		*N*	%	*N*	%		
Single	HPV 16	230	75.7	226	68.3	9.213	0.511
	HPV 18	30	9.9	39	11.8		
	HPV 33	12	3.9	14	4.2		
	HPV 58	10	3.3	14	4.2		
	HPV 31	6	2.0	6	1.8		
	HPV 45	5	1.6	5	1.5		
	HPV 52	1	0.3	7	2.1		
	HPV 59	3	1.0	6	1.8		
	HPV 73	1	0.3	3	0.9		
	Other single HR-HPV	6	2.0	10	3.0		
	Other single LR-HPV	0	0.0	1	0.3		
	Total	304	100	331	100		
Multiple	HPV16 + 6	4	3.6	6	9.4	14.378	0.265
	HPV16 + 18	29	25.9	9	14.1		
	HPV16 + 33	11	9.8	3	4.7		
	HPV16 + 31	4	3.6	2	3.1		
	HPV16 + 45	6	5.4	4	6.2		
	HPV16 + 52	5	4.5	3	4.7		
	HPV16 + 58	13	11.6	9	14.1		
	HPV16 + 59	5	4.5	3	4.7		
	HPV 16 + 73	2	1.8	4	6.2		
	HPV16 + others (2types)	10	8.9	11	17.2		
	HPV18 + others (2types)	6	5.4	3	4.7		
	HPV other 2 types	6	5.4	5	7.8		
	HPV 3 types	11	9.8	2	3.1		
	Total	112	100.0	64	100.0		

^*^Fisher’s Exact Test; HPV16 was the most frequent type in both single-type and multiple-type HPV infections within the CCTS. There was no statistical difference in HPV Genotyping distribution rate between the two assays.

**Figure 1:**
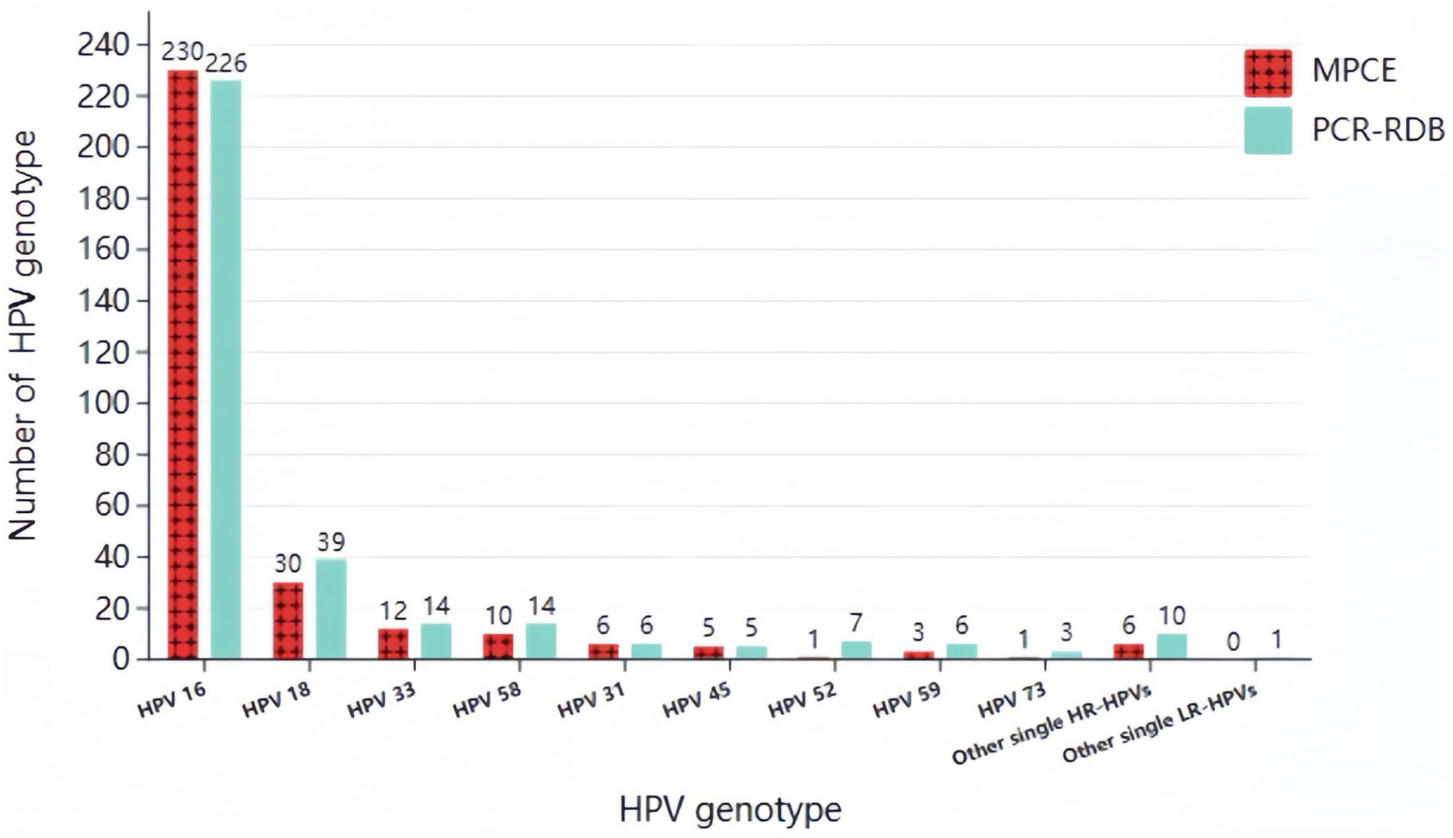
Prevalence of single-type HPV infection detected by two assays in HPV positive CCTS.

**Figure 2:**
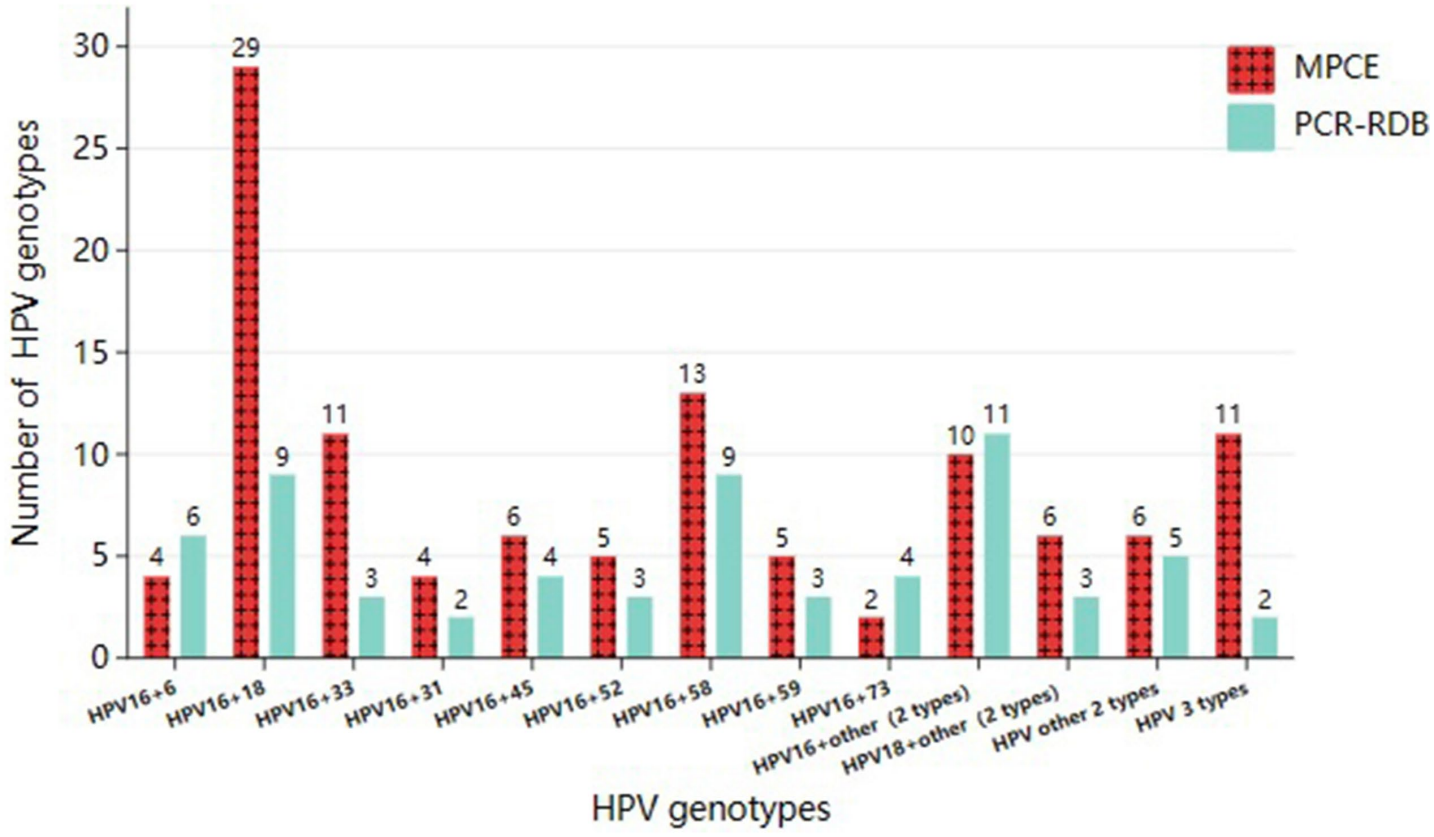
Prevalence of multiple-type HPV infections detected by two assays in HPV positive CCTS.

In the MPCE assay, among the total 416 HPV-positive detections, single and multiple infections accounted for 73.1% (304/416) and 26.9% (112/416), respectively. Among the 112 cases with multiple infections, HPV16 + 18 (25.9%, 29/112) was the most common genotype combination, followed by HPV16 + 58 (11.6%, 13/112) and HPV16 + 33 (9.8%, 11/112; Figures 1, 2).

In the PCR-RDB assay, among the 395 HPV-positive detections, single and multiple infections accounted for 83.8% (331/395) and 16.2% (64/395), respectively. Among the 64 cases with multiple infections, HPV16 + 18 (14.1%, 9/64) and HPV16 + 58 (14.1%, 9/64) were the most common genotype combinations, followed by HPV16 + 6 (9.4%, 6/64; Figures 1, 2).

Despite the differences between both assays, HPV16 was the most frequent type in single-type and multiple-type HPV infections within the CCTS. Furthermore, no statistical difference was observed in the HPV Genotyping distribution rate between both assays.

### 3.3 Comparison of positive rates of single-type and multiple-type HPV DNA genotyping infections across different age groups between the two detection assays

Among all the detected positive cases in both assays, in the population over 50 years old, the HPV multiple-type infection rate detected via the MPCE assay was significantly higher than that detected by the PCR-RDB assay, with statistical significance (*p* = 0.002). This indicates that in the age group over 50 years old within the CCTS, the MPCE detection assay demonstrates a higher proportion of detecting multiple HPV infections (Table 4).

**Table 4:** Comparison between single and multiple HPV infections in different age groups.

Age	MPCE		PCR-RDB		χ2 test	*P-*value
	Single-type	Multiple-type	Single-type	Multiple-type		
	*N* (%)	*N* (%)	*N* (%)	*N* (%)		
≤50	111(74.5)	38(25.5)	119(83.8)	23(16.2)	3.801	0.051
>50	193(72.3)	74(27.7)	212(83.8)	41(16.2)	9.991	0.002*
Total	304	112	331	64		

^*^In the age group over 50 years old within the CCTS, the MPCE assay demonstrated a higher proportion of detecting multiple HPV infections.

## 4 Discussion

HPV was identified in 99.7% of patients with cervical cancer (25). Persistent infection with HR HPV is the primary cause of pre-cancerous lesions leading to cervical cancer. HPV genes encode three early proteins: E5, E6, and E7. The E5 protein has a relatively weak transforming effect but activates membrane-associated protein kinases. E6 and E7 are viral oncogenes that regulate the expression and replication of viral genes in host cells. The E6 protein binds to p53, while the E7 protein binds to the Rb protein, thereby inactivating these tumor suppressor genes and enabling unlimited cell growth (11, 26, 27). The oncoproteins encoded by the E6 and E7 genes are crucial in cervical epithelial carcinogenesis. In the early stages up to CIN I, HPV primarily exists freely within host cells. As the lesion advances, the HPV genome integrates into the host cell genome. As the lesion severity increases, HPV eventually exists in an integrated state. During integration, the E6/E7 genes persist while the L1 gene may be lost, posing a risk of false-negative detection results. Most PCR-based tests only amplify the L1 region of the virus. Therefore, PCR false negatives may occur due to the loss of this region during viral integration. In contrast, evaluating E6/E7 mRNA expression can indicate high-grade lesions or cervical cancer, as E6/E7 mRNA levels increase after the viral genome integrates into host cells (28).

In this study, CCTS from patients diagnosed through pathological histology was assessed, providing a more direct validation of the association between cervical cancer and HPV infection than traditional cytological testing. However, both assays were effective for HPV DNA genotyping. The overall positivity rate of HPV detection with the MPCE assay was higher than that with the PCR-RDB assay, with *p* < 0.05 indicating a statistically significant difference between the two assays. Using the oncogenic genes E6/E7 DNA as detection targets reduced the missed detection rate in CCTS by approximately 5.0% compared to that targeting the L1 gene. Another study produced similar results. A comparison of detection using universal primers for the L1 region and typing primers for E6/E7 in 56 biopsy samples of invasive cervical cancer tissue revealed that 23 samples exhibited L1 region deletions (29). For cervical cancer, a malignancy with a well-defined pathogenic mechanism, higher detection rates of oncogenic HPV facilitate earlier identification of potential patients.

The genotyping results of positive HPV DNA detected through the two testing assays were more significant in terms of understanding the etiology. Our study revealed that the MPCE assay targeting the oncogenic genes E6/E7 DNA detected a higher rate of multiple-type infections in CCTS of patients over 50 years old. A study indicated that E6/E7 target regions are more sensitive than L1 (30). While the time of HPV infection in patients was unknown based on the process of HPV integration into host cells, we hypothesize that without intervention, as time progresses from HPV infection to cervical cell infiltration and eventual carcinogenesis, the increasing severity of persistent HPV infection makes E6/E7 fragments easier to detect in host cells than that of L1 fragments. This suggests that detection based on L1 fragments poses a risk of false-negative results. Therefore, PCR tests may yield false negatives owing to the loss of L1 fragments during viral integration. This may suggest that in older individuals with long-term HR-HPV infections, targeting HPV oncogenic genes E6/E7 are more effective for detection. One study showed that multiple HPV infections exerted a synergistic effect on cervical cancer development (31). One study found that multiple HPV genotype infections correlate with poorer cervical cancer survival than that of single genotype infection (32). The presence of multiple HPV genotypes may extend the duration of persistent HPV infection and increase the risk of carcinogenesis.

Table 3 shows a high degree of overlap in the top high-frequency HR genotype distribution detected through the two assays, corresponding to the seven HR-HPV through the 9-valent HPV vaccine (HPV 16/18/31/33/45/52/58). This suggests that vaccinating large populations of women in the appropriate age group with the 9-valent HPV vaccine can significantly reduce the risk of cervical cancer, ultimately aiming to reduce or even eliminate the disease. The HPV vaccination rate in China remains relatively low (33, 34). While the HPV vaccine is not yet incorporated into the national immunization program, qualified regions should be encouraged to conduct trials.

Pathological diagnosis of cervical cancer tissue is considered the gold standard for diagnosing cervical cancer, although there is not a 100% definitive causal relationship between HPV infection and cervical cancer. The epidemiological study revealed that almost all cases of cervical cancer are caused by HPV. However, approximately 5% of tumors are not associated with HPV-persistent infection. HPV-negative status can arise from various scenarios: (1) HPV-independent cancers (true negative), such as certain adenocarcinoma subtypes and a few cases of squamous carcinoma (4); (2) loss of the HPV genome during integration; (35); (3) presence of viral genotypes not included in the molecular tests; (4) failure of the detection of the diagnostic method employed; or (5) misclassification of cancers as primary cervical, including metastases or primary uterine corpus neoplasms.

In this study, the 22 samples that tested positive with the MPCE assay but negative with the PCR-RDB assay require further investigation in future research. This discrepancy is most likely attributable to the absence of the L gene, which led to the failure of the PCR-RDB assay to detect HPV. In future studies, we plan to expand the sample size to explore the rate of L gene loss during cervical cancer progression, its integration with host genes, and the timeframe of this occurrence. This could potentially provide significant insights for detecting and treating cervical cancer. In contrast, there were eight cases where neither assay detected HPV infection. One possibility is that the testing kits did not include specific HPV DNA types, leading to undetected results. Another possibility is true HPV negativity, indicating no HPV infection was present. Due to less focus on HPV-negative cervical cancer, the clinical significance of HPV-negative cervical cancer may have been underestimated. Studies suggest that HPV-negative cancer may exhibit worse clinical features and distinct biological characteristics than that of HPV-positive cervical cancer (36, 37). Some scholars advocate combining HPV genotyping with cytological evaluation in co-testing to mitigate the risk of false-negative results caused by associated with a single HPV genotyping (38). In 2020, the WHO updated the Female Genital Tumors classification (5th edition) and recognized that a proportion of cervical cancers, particularly adenocarcinomas, may not be linked to HPV infection (39). This area also warrants further focused research and attention.

Quality control of the kit significantly influences the reliability of the test. Both MPCE and PCR-RDB incorporate internal control to ensure successful DNA extraction and amplification steps. Nevertheless, the implementation of the dUTP-UNG system to prevent carry-over contamination, along with the inclusion of dual internal controls and the capacity to detect and genotype 25 HPV types simultaneously in a single reaction, as well as highly automated sample analysis process of the MPCE assay, have enabled its widespread application in clinical settings.

Our study had some limitations. This study focused solely on evaluating and comparing the performance of the newly developed MPCE assay with the commonly used PCR-RDB. We did not analyze common screening indicators such as sensitivity and specificity. As the progression from HPV infection to cervical cancer is a relatively lengthy process, these indicators will be further analyzed and validated in subsequent large-scale population screenings in common people. During that period, we will focus on screening precancerous lesions, conducting staged studies on lesions, and providing reliable theoretical support for reducing cervical cancer incidence through effective detection methods and clear intervention measures. While cervical cancer is primarily associated with high-risk HPV infections, approximately 5% of cervical cancers are classified as true HPV-negative cervical cancers. This highlights the inherent complexity of cervical cancer. Here, we focused on samples diagnosed with cervical cancer based on histology, which does not entirely rule out cancer risk from other causes. The setting of the gold standard has certain limitations. However, it is undeniable that when appropriate experiments are selected for large-scale population screening, the benefits of early detection, diagnosis, and treatment of cervical cancer precursor lesions far outweigh the risks of missed diagnoses. Non-invasive examination methods and the detection of fluid biomarkers, applied in large-scale population screening, can significantly benefit the early detection, diagnosis, and treatment of cervical cancer precursor lesions.

However, further research necessitates increasing the sample size and conducting comparative testing on a larger sample of pathological tissues. Targeting E6/E7 genes instead of the L1 gene can mitigate false-negative results from HPV genome integration into the human genome. Therefore, the oncogenic genes E6/E7 are more suitable targets for HPV DNA detection.

Given the large population in China, uneven distribution of healthcare resources, and relatively low rates of cervical cancer screening and vaccination, adopting an HPV screening plan that aligns better with the national conditions in China to achieve early detection, diagnosis, and treatment of cervical cancer is crucial. Considering the screening value, economic cost of the detection assay, and healthcare resource allocation in China, adopting MPCE assay as one of the primary methods for population-based cervical cancer screening is recommended. A screening interval of 5 years should be established, along with the maintenance of corresponding records. Special attention should be given to the distribution of HPV genotypes among populations with precancerous lesions. The MPCE assay proposed and validated in this study can use cervical exfoliated cell samples as research specimens in practical screening efforts to identify more patients early, resulting in superior cost-effectiveness.

## Data availability statement

The original contributions presented in the study are included in the article/Supplementary material, further inquiries can be directed to the corresponding author.

## Ethics statement

This study was approved by the Ethics Committee of Nanjing Traditional Chinese Medicine Hospital (approval no. 2012NJL008). The studies were conducted in accordance with the local legislation and institutional requirements. The participants provided their written informed consent to participate in this study. Written informed consent was obtained from the individual(s) for the publication of any potentially identifiable images or data included in this article.

## Author contributions

LQ: Writing – review & editing, Writing – original draft, Visualization, Validation, Supervision, Software, Resources, Project administration, Methodology, Investigation, Funding acquisition, Formal analysis, Data curation, Conceptualization. DL: Writing – review & editing, Validation, Supervision, Project administration, Methodology, Investigation, Funding acquisition, Data curation. ZW: Writing – review & editing, Resources, Methodology, Data curation. JL: Writing – review & editing, Resources, Methodology, Data curation. CH: Writing – review & editing, Resources, Methodology, Data curation. JM: Writing – review & editing, Resources, Methodology, Data curation. JG: Writing – review & editing, Visualization, Validation, Supervision, Software, Resources, Project administration, Methodology, Investigation, Funding acquisition, Formal analysis, Data curation, Conceptualization.

## References

[1] Sung H Ferlay J Siegel RL Laversanne M Soerjomataram I Jemal A. Global Cancer statistics 2020: GLOBOCAN estimates of incidence and mortality worldwide for 36 cancers in 185 countries. CA Cancer J Clin. (2021) 71:209–49. doi: 10.3322/caac.21660 33538338

[2] Tota JE Chevarie-Davis M Richardson LA Devries M Franco EL. Epidemiology and burden of HPV infection and related diseases: implications for prevention strategies. Prev Med. (2011) 53:S12–21. doi: 10.1016/j.ypmed.2011.08.017, PMID: 21962466

[3] Schiffman M Doorbar J Wentzensen N de Sanjosé S Fakhry C Monk BJ. Carcinogenic human papillomavirus infection. Nat Rev Dis Prim. (2016) 2:16086. doi: 10.1038/nrdp.2016.86 27905473

[4] Cancer Genome Atlas Research Network, Albert Einstein College of Medicine, Analytical Biological Services. Integrated genomic and molecular characterization of cervical cancer. Nature. (2017) 543:378–84. doi: 10.1038/nature21386, PMID: 28112728 PMC5354998

[5] World Health Organization. WHO guideline for screening and treatment of cervical pre-cancer lesions for cervical cancer prevention: use of mRNA tests for human papillomavirus (HPV) [EB/OL]. (2022). Available at: https://www.who.int/publications/i/item/9789240040434 (Accessed 11 November 2022). 35044737

[6] Cervical Cancer Treatment Guidelines Available at: http://www.nhc.gov.cn/yzygj/s7659/202204/a0e67177df1f439898683e1333957c74/files/0feefc11d98840898b136ac3d9a4ee20.pdf (2022). (Accessed 11 April 2022).

[7] Bedell SL Goldstein LS Goldstein AR Goldstein AT. Cervical Cancer screening: past, present, and future. Sex Med Rev. (2020) 8:28–37. doi: 10.1016/j.sxmr.2019.09.005, PMID: 31791846

[8] Bell M Verberckmoes B Devolder J Vermandere H Degomme O Guimarães YM. Comparison between the Roche Cobas 4800 human papillomavirus (HPV), Abbott RealTime high-risk HPV, Seegene Anyplex II HPV28, and novel Seegene Allplex HPV28 assays for high-risk HPV detection and genotyping in mocked self-samples. Microbiol Spectr. (2023) 11:e0008123. doi: 10.1128/spectrum.00081-23, PMID: 37284753 PMC10433804

[9] Martinez-Zapien D Ruiz FX Poirson J Mitschler A Ramirez J Forster A. Structure of the E6/E6AP/p53 complex required for HPV-mediated degradation of p53. Nature. (2016) 529:541–5. doi: 10.1038/nature16481, PMID: 26789255 PMC4853763

[10] Pal A Kundu R. Human papillomavirus E6 and E7: the cervical Cancer hallmarks and targets for therapy. Front Microbiol. (2020) 10:3116. doi: 10.3389/fmicb.2019.03116, PMID: 32038557 PMC6985034

[11] Della Fera AN Warburton A Coursey TL Khurana S McBride AA. Persistent human papillomavirus infection. Viruses. (2021) 13:321. doi: 10.3390/v13020321, PMID: 33672465 PMC7923415

[12] Arroyo Mühr LS Lagheden C Hassan SS Eklund C Dillner J. The international human papillomavirus reference center: standardization, collaboration, and quality assurance in HPV research and diagnostics. J Med Virol. (2023) 95:e29332. doi: 10.1002/jmv.29332, PMID: 38115556

[13] Bhatla N Singhal S. Primary HPV screening for cervical cancer. Best Pract Res Clin Obstet Gynaecol. (2020) 65:98–108. doi: 10.1016/j.bpobgyn.2020.02.008 32291178

[14] Tumban E. A current update on human papillomavirus-associated head and neck cancers. Viruses. (2019) 11:922. doi: 10.3390/v11100922, PMID: 31600915 PMC6833051

[15] Wu RF Dai M Qiao YL Clifford GM Liu ZH Arslan A. Human papillomavirus infection in women in Shenzhen City, People's Republic of China, a population typical of recent Chinese urbanisation. Int J Cancer. (2007) 121:1306–11. doi: 10.1002/ijc.22726, PMID: 17417776

[16] Kocjan BJ Hošnjak L Poljak M. Detection of alpha human papillomaviruses in archival formalin-fixed, paraffin-embedded (FFPE) tissue specimens. J Clin Virol. (2016) 76:S88–97. doi: 10.1016/j.jcv.2015.10.007, PMID: 26514313

[17] Santesso N Mustafa RA Schünemann HJ Arbyn M Blumenthal PD Cain J. Guideline support group. World Health Organization guidelines for treatment of cervical intraepithelial neoplasia 2-3 and screen-and-treat strategies to prevent cervical cancer. Int J Gynaecol Obstet. (2016) 132:252–8. doi: 10.1016/j.ijgo.2015.07.038, PMID: 26868062

[18] Xia C Xu X Zhao X Hu S Qiao Y Zhang Y. Effectiveness and cost-effectiveness of eliminating cervical cancer through a tailored optimal pathway: a modeling study. BMC Med. (2021) 19:62. doi: 10.1186/s12916-021-01930-9, PMID: 33653331 PMC7927373

[19] Zhang J Zhao Y Dai Y Dang L Ma L Yang C. Effectiveness of high-risk human papillomavirus testing for cervical Cancer screening in China: a multicenter, open-label, Randomized Clinical Trial. JAMA Oncol. (2021) 7:263–70. doi: 10.1001/jamaoncol.2020.6575, PMID: 33377903 PMC7774051

[20] Poljak M Kocjan BJ Oštrbenk A Seme K. Commercially available molecular tests for human papillomaviruses (HPV): 2015 update. J Clin Virol. (2016) 76:S3–S13. doi: 10.1016/j.jcv.2015.10.023, PMID: 26601820

[21] Arbyn M Snijders PJ Meijer CJ Berkhof J Cuschieri K Kocjan BJ. Which high-risk HPV assays fulfil criteria for use in primary cervical cancer screening? Clin Microbiol Infect. (2015) 21:817–26. doi: 10.1016/j.cmi.2015.04.015, PMID: 25936581

[22] de Sanjose S Holme F. What is needed now for successful scale-up of screening? Papillomavirus Res. (2019) 7:173–5. doi: 10.1016/j.pvr.2019.04.011, PMID: 31002883 PMC6477512

[23] Schiffman M de Sanjose S. False positive cervical HPV screening test results. Papillomavirus Res. (2019) 7:184–7. doi: 10.1016/j.pvr.2019.04.012, PMID: 31029852 PMC6514435

[24] Hanby AM Walker C. Tavassoli FA, Devilee P: Pathology and genetics: Tumours of the breast and female genital organs. WHO classification of Tumours series—volume IV. Lyon, France: IARC Press. Breast Cancer Res. (2004) 6:133. doi: 10.1186/bcr788

[25] Walboomers JM Jacobs MV Manos MM Bosch FX Kummer JA Shah KV. Human papillomavirus is a necessary cause of invasive cervical cancer worldwide. J Pathol. (1999) 189:12–9. doi: 10.1002/(SICI)1096-9896(199909)189:1<12::AID-PATH431>3.0.CO;2-F 10451482

[26] Harden ME Munger K. Human papillomavirus molecular biology. Mutat Res Rev Mutat Res. (2017) 772:3–12. doi: 10.1016/j.mrrev.2016.07.002, PMID: 28528688 PMC5500221

[27] Doorbar J Egawa N Griffin H Kranjec C Murakami I. Human papillomavirus molecular biology and disease association. Rev Med Virol. (2015) 25:2–23. doi: 10.1002/rmv.1822, PMID: 25752814 PMC5024016

[28] Xing B Guo J Sheng Y Wu G Zhao Y. Human papillomavirus-negative cervical Cancer: a comprehensive review. Front Oncol. (2021) 10:606335. doi: 10.3389/fonc.2020.606335, PMID: 33680928 PMC7925842

[29] Karlsen F Kalantari M Jenkins A Pettersen E Kristensen G Holm R. Use of multiple PCR primer sets for optimal detection of human papillomavirus. J Clin Microbiol. (1996) 34:2095–100. doi: 10.1128/jcm.34.9.2095-2100.1996, PMID: 8862564 PMC229196

[30] Vaughan LM Malinowski DP. Comments on: limitations of HPV DNA testing in screening of cervical adenocarcinomas. Rev Bras Ginecol Obstet. (2019) 41:357–9. doi: 10.1055/s-0039-1688710, PMID: 31181587

[31] Trottier H Mahmud S Costa MC Sobrinho JP Duarte-Franco E Rohan TE. Human papillomavirus infections with multiple types and risk of cervical neoplasia. Cancer Epidemiol Biomarkers Prev. (2006) 15:1274–80. doi: 10.1158/1055-9965.EPI-06-0129 16835323

[32] Nogueira Dias Genta ML Martins TR Mendoza Lopez RV Sadalla JC de Carvalho JPM Baracat EC. Multiple HPV genotype infection impact on invasive cervical cancer presentation and survival. PLoS One. (2017) 12:e0182854. doi: 10.1371/journal.pone.0182854, PMID: 28829791 PMC5567480

[33] Yuan K Zhang P Yang M. Vaccination prevalence and influencing factors of HPV vaccine among women in Tengzhou city, 2018-2020: a paired case-control study. Chin J Public Health. (2021) 37:1746–50. doi: 10.11847/zgggws1134989

[34] Liu J Wu L Bai Q Ren J Shao H Huang Z. Surveillance for coverage of human papillomavirus (HPV) vaccine and adverse events following immunization with HPV vaccine in Shanghai, 2017-2019. Chin J Vaccines Immun. (2020) 26:322–48.

[35] Tjalma W. HPV negative cervical cancers and primary HPV screening. Facts Views Vis Obgyn. (2018) 10:107–13. PMID: 31110650 PMC6516188

[36] Barreto CL Martins DB de Lima Filho JL Magalhães V. Detection of human papillomavirus in biopsies of patients with cervical cancer, and its association with prognosis. Arch Gynecol Obstet. (2013) 288:643–8. doi: 10.1007/s00404-013-2803-2 23529684

[37] Lee JE Chung Y Rhee S Kim TH. Untold story of human cervical cancers: HPV-negative cervical cancer. BMB Rep. (2022) 55:429–38. doi: 10.5483/BMBRep.2022.55.9.042, PMID: 35725012 PMC9537028

[38] Brakebill A Morgan A Lieberman RW. Primary HPV screening vs Cotesting for cervical Cancer. JAMA. (2023) 330:2121. doi: 10.1001/jama.2023.20370, PMID: 38051332

[39] Höhn AK Brambs CE Hiller GGR May D Schmoeckel E Horn LC. 2020 WHO classification of female genital tumors. Geburtshilfe Frauenheilkd. (2021) 81:1145–53. doi: 10.1055/a-1545-4279, PMID: 34629493 PMC8494521

