# Supplementary material for: Comparison of multiplex PCR capillary electrophoresis assay and PCR-reverse dot blot assay for human papillomavirus DNA genotyping detection in cervical cancer tissue specimens
Source: Front Public Health. 2024 Jul 19;12:1421774. doi: 10.3389/fpubh.2024.1421774 (PMC11294082; doi:10.3389/fpubh.2024.1421774)
Supplement: Supplementary file 1 [file Table_1.DOCX]

Supplementary Material

Comparison of Multiplex PCR Capillary Electrophoresis Assay and PCR-Reverse Dot Blot Assay for Human Papillomavirus DNA Genotyping Detection in Cervical Cancer Tissue Specimens

Lei Qin^1^, Dan Li^1^, Zhihui Wang^2^, Jianyun Lan^3^, Chunrong Han^4^, Jing Mei^5^, Jianxiang Geng^1*,6^

^1^ Nanjing Leiyue Clinical Laboratory, Jiangsu, China

^2^ Department of Pathology, Linyi Cancer Hospital, Shangdong, China

^3^ Department of Pathology, Yancheng First People’s Hospital, Jiangsu, China

^4^ Department of Pathology, Nanjing Lishui District People’s Hospital, Jiangsu, China

^5^ Department of Pathology, People’s Hospital of Dangtu, Anhui, China

^6^ The Cross-Strait Precision Medicine Association HPV Infection Disease Professional Committee, Nanjing, China

*** Correspondence: Jianxiang Geng**Corresponding Author
njgjx2022@163.com

# Supplementary Table 1. The list of the specimen source of four hospitals in this study

| **No.** | **Name of Hospitals** | **City** | **Province** |
| --- | --- | --- | --- |
| 1 | Linyi Cancer Hospital | Linyi | Shandong |
| 2 | Yancheng First People’s Hospital | Yancheng | Jiangsu |
| 3 | Nanjing Lishui District People’s Hospital | Nanjing | Jiangsu |
| 4 | People’s Hospital of Dangtu | Ma'anshan | Anhui |
